# Leishmaniasis Recidivans in Rural Venezuela

**DOI:** 10.5826/dpc.1003a62

**Published:** 2020-06-29

**Authors:** Ariadna Perez Sanchez, Rajani Katta

**Affiliations:** 1 Department of Internal Medicine, The University of Texas Health Science Center, San Antonio, TX, USA; 2 Department of Dermatology, McGovern Medical School, Houston, TX, USA

**Keywords:** leishmaniasis recidivans, *Leishmania braziliensis*, localized cutaneous leishmaniasis, ulceration, antimonials

## Clinical Presentation

**Figure 1:**
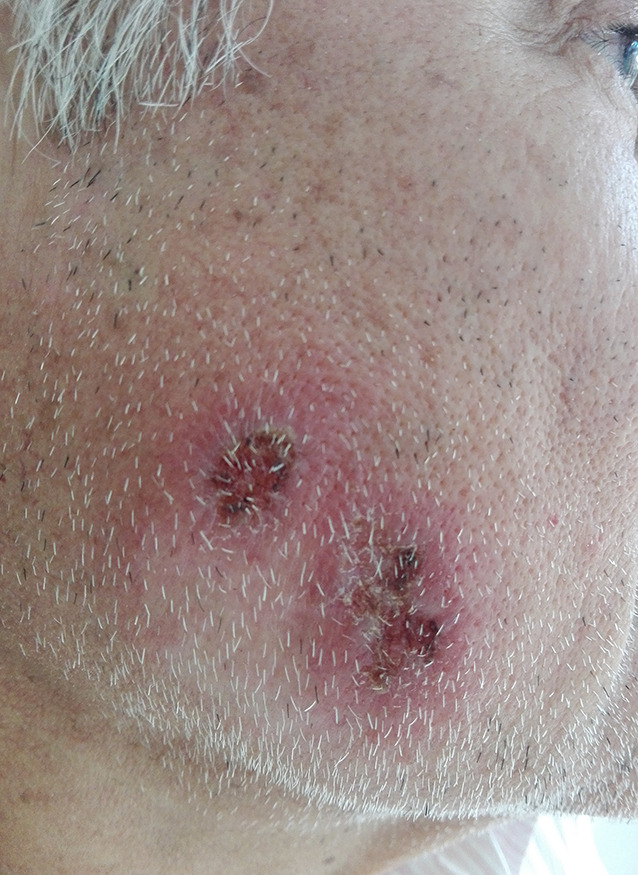
Two ulcerations on the right cheek, overlying scar from previous infection.

A 66-year-old man presented with 2 asymptomatic ulcerations on the right cheek (Figure 1). These began as enlarging papules that ulcerated 2 weeks later. He had been treated for leishmaniasis a year previously, with an ulcer in the same location. Diagnosis had been made by skin biopsy and polymerase chain reaction, identifying *Leishmania braziliensis*. At that time, he was given intramuscular meglumine antimoniate; his lesions had healed completely within 1 month.

## Teaching Point

Leishmaniasis recidivans (LR) is a rare presentation of localized cutaneous leishmaniasis, recurring at the site of a previously healed ulcer [1], as in this case. It typically affects the face, often the cheek. *Leishmania braziliensis* is one of the species linked to New World LR. While the pathogenesis of LR is not known, risk factors include parasite resistance and incomplete treatment. Resistance to antimonials is increasingly a concern in some regions [2].

## References

[1] Masood S, Naveed S, Alvi RU (2012). Infiltrated leishmaniasis recidivans cutis on the face: a rare clinical presentation. Trop Doct.

[2] Ponte-Sucre A, Gamarro F, Dujardin JC (2017). Drug resistance and treatment failure in leishmaniasis: a 21st century challenge. PLoS Negl Trop Dis.

